# Gene Fusions Derived by Transcriptional Readthrough are Driven by Segmental Duplication in Human

**DOI:** 10.1093/gbe/evz163

**Published:** 2019-08-10

**Authors:** Ann M McCartney, Edel M Hyland, Paul Cormican, Raymond J Moran, Andrew E Webb, Kate D Lee, Jessica Hernandez-Rodriguez, Javier Prado-Martinez, Christopher J Creevey, Julie L Aspden, James O McInerney, Tomas Marques-Bonet, Mary J O’Connell

**Affiliations:** 1 Bioinformatics and Molecular Evolution Group, School of Biotechnology, Dublin City University, Ireland; 2 Computational and Molecular Evolutionary Biology Group, School of Biology, Faculty of Biological Sciences, The University of Leeds, United Kingdom; 3 Institute for Global Food Security, Queens University Belfast, United Kingdom; 4 Teagasc Animal and Bioscience Research Department, Animal & Grassland Research and Innovation Centre, Teagasc, Grange, Dunsany, County Meath, Ireland; 5 School of Biological Sciences, University of Auckland, New Zealand; 6 School of Fundamental Sciences, Massey University, New Zealand; 7 Institute of Evolutionary Biology (UPF-CSIC), PRBB, Dr. Aiguader 88, 08003 Barcelona, Spain; 8 Wellcome Trust Sanger Institute, Wellcome Trust Genome Campus, Hinxton, United Kingdom; 9 Institute of Biological, Environmental and Rural Sciences, Aberystwyth University, United Kingdom; 10 School of Molecular and Cellular Biology, Faculty of Biological Sciences, The University of Leeds, United Kingdom; 11 Division of Evolution and Genomic Sciences, School of Biological Sciences, Faculty of Biology, Medicine and Health, The University of Manchester, M13 9PL, United Kingdom; 12 School of Life Sciences, Faculty of Medicine and Health Sciences, The University of Nottingham, NG7 2RD, United Kingdom; 13 Catalan Institution of Research and Advanced Studies (ICREA), Passeig de Lluís Companys, 23, 08010, Barcelona, Spain; 14 NAG-CRG, Centre for Genomic Regulation (CRG), Barcelona Institute of Science and Technology (BIST), Baldiri i Reixac 4, 08028 Barcelona, Spain; 15 Institut Català de Paleontologia Miquel Crusafont, Universitat Autònoma de Barcelona, Edifici ICTA-ICP, c/ Columnes s/n, 08193 Cerdanyola del Vallés, Barcelona, Spain

**Keywords:** sequence similarity networks, novel genes, segmental duplication, mechanisms of protein-coding evolution, Great Ape Comparative genomics, transcriptional readthrough

## Abstract

Gene fusion occurs when two or more individual genes with independent open reading frames becoming juxtaposed under the same open reading frame creating a new fused gene. A small number of gene fusions described in detail have been associated with novel functions, for example, the hominid-specific *PIPSL* gene, *TNFSF12*, and the *TWE-PRIL* gene family. We use Sequence Similarity Networks and species level comparisons of great ape genomes to identify 45 new genes that have emerged by transcriptional readthrough, that is, transcription-derived gene fusion. For 35 of these putative gene fusions, we have been able to assess available RNAseq data to determine whether there are reads that map to each breakpoint. A total of 29 of the putative gene fusions had annotated transcripts (9/29 of which are human-specific). We carried out RT-qPCR in a range of human tissues (placenta, lung, liver, brain, and testes) and found that 23 of the putative gene fusion events were expressed in at least one tissue. Examining the available ribosome foot-printing data, we find evidence for translation of three of the fused genes in human. Finally, we find enrichment for transcription-derived gene fusions in regions of known segmental duplication in human. Together, our results implicate chromosomal structural variation brought about by segmental duplication with the emergence of novel transcripts and translated protein products.

## Introduction

The emergence of novel protein*-*coding gene families in animal genomes has been widely studied from a number of perspectives and phylogenetic depths (Kaessmann 2010; Dunwell et al. 2017; Villanueva-Cañas et al. 2017; Paps and Holland 2018). There are many mechanisms of novel gene genesis that have been elucidated thus far, and they include de novo genesis from noncoding DNA, retrotransposition, domain/exon shuffling, mobile elements, noncoding RNA, reading-frame shifts, gene duplication, and gene fusion/fission among others (Long et al. 2003). The emergence of new genes has been associated with the emergence of novel functions and phenotypes through the animal kingdom and beyond. For example, independently in both mammals and in a viviparous lizard, new genes of viral origin derived by retrotransposition have been shown to be essential for placentation (Lee et al. 2000; Cornelis et al. 2017). Domain shuffling has contributed significantly to the evolution of vertebrate-specific features such as the evolution of cartilage, craniofacial structures, and adaptive immune system (Kawashima et al. 2009). Duplication (from whole genome duplication to the duplication of an individual gene) has contributed widely to the evolution of novel protein*-*coding genes and this mechanism has had profound effects on the evolution of complexity and diversity of life (Ohno et al. 1968; Ohno 1970; Crow and Wagner 2006).

Of course, these mechanisms are not mutually exclusive and can work in combination to produce new genes, a classic example of which is *jingwei*—a processed functional protein*-*coding gene (Long and Langley 1993). *Jingwei* originated ∼2 Million Years Ago (mya) in African Drosophila species by gene duplication (of the *yande* gene) and retrotransposition (of the *Adh* gene) to produce a fused gene that underwent intense positive selection, has preferences for long-chain primary alcohols, and has a testis-specific expression pattern (Long and Langley 1993; Zhang et al. 2004). Overall, these and other studies suggest that *jingwei* has evolved a new function for hormone and pheromone biosynthesis/degradation processes in Drosophila (Zhang et al. 2004).

Gene fusion can be achieved by transcription mediated processes such as the readthrough of adjacent genes to produce a novel transcript, we refer to these as transcription-derived gene fusion (TDGFs). Alternatively, gene fusion can occur by a variety of structural rearrangements such as gene duplication and reinsertion into (or adjacent to) another coding sequence resulting in a genome encoded fusion event, we refer to these as DNA-mediated gene fusions (Kaessmann 2010; Latysheva et al. 2016). From detailed studies of a small number of fused genes, we know they do not necessarily have to follow the same expression profile as their parents thereby bringing existing functionality to novel tissues and subcellular locations, and indeed their functions are not simply additive of their parents (Thomson et al. 2000; Pradet-Balade et al. 2002; Akiva et al. 2005; Parra et al. 2005). For example, the *PIP5K1A* gene is shared among hominoids and was formed by TDGF followed by retrotransposition. In comparison to its parents, *PIP5K1A* has a testes-specific expression pattern and has undergone positive selection and a substrate affinity shift (Babushok et al. 2007).

For two or more genes to merge by TDGF and become a single transcript and potentially a single protein product, the parent genes must occupy a reasonably close position on a given chromosome. Many structural rearrangement processes exist that can bring about close proximity of genes on a genome, for example, inversion, insertion, deletion, translocation, and segmental duplication (SD). SD (also known as low copy repeats) are duplicates of 1–5 kb in length and remain >90% similar to that of the original sequence. Interestingly, while the overall rate of genomic rearrangement reduced in hominids, the rate of SD increased in the Great Ape clade (Marques-Bonet, Girirajan, et al. 2009; Marques-Bonet, Kidd, et al. 2009). In addition, in human, it has been shown that some regions of SD are enriched for protein*-*coding genes (Lorente-Galdos et al. 2013), data from other great apes are slowly emerging and chimpanzee (hominoid) seems to follow a similar trend (Cheng et al. 2005). Regions of SD tend to cluster near the peri-centromeric or peri-telomeric regions of chromosomes (Feng et al. 2017) and form complex clusters due to formation of duplication hotspots at regions of genomic instability (Ji et al. 2000; Samonte and Eichler 2002; Armengol et al. 2003). Therefore, it is proposed that genomic instability brought about by increased gene rich SD activity in the great ape clade may contribute to the emergence of novel protein*-*coding regions by, for example, exon shuffling and/or gene fusion (Bailey et al. 2002; Akiva et al. 2005; Denoeud et al. 2007). Indeed, it has been shown that the reshuffling of genes inside SD regions of hominid genomes led to the formation of an abundance of mosaic gene structures across these species but until now it has been unclear whether these novel structures produce novel transcripts and protein products (Bailey et al. 2002; She et al. 2004; Marques-Bonet, Girirajan et al. 2009).

In this article, we set out to determine those gene families that have arisen by TDGF across a data set of human, five nonhuman primates, and mouse, using sequence similarity networks (SSNs). SSNs are undirected bipartite graphs based on sequence similarity searches whereby an edge is drawn between two or more nodes (genes) only if they contain sequence similarity above a user-defined threshold namely either a percentage identity or e-value (Jachiet et al. 2013). We employ deconstruction techniques to deconstruct global SSNs into nontransitive triplets, or fusion gene families (Berry et al. 2010). After the identification of TDGFs across the data set, we investigate and cross compare their transcriptional and translational profiles across each species and to nonfused protein*-*coding genes in the same species. We also assess the ability of TDGFs to acquire alternative splice isoforms (Wang et al. 2015). Previous investigations of new genes have revealed a trend toward testes-specific expression (Kaessmann 2010), by obtaining transcriptional profiles TDGF expression can be compared with those of new genes generated by alternative mechanisms. To assess TDGF expression across the data set, we perform a metadata analysis of RNA sequencing (Brawand et al. 2011) data for all seven species across a panel of six tissues (brain, cerebellum, kidney, heart, liver, and testis) and we complement this with novel RT-qPCR data we generated for human across a panel of five tissues (liver, brain, placenta, lung, and testis) and splice factor (SF) binding analysis. To investigate TDGF translational profiles, we use four ribosequencing data sets across three human cell types (fibroblast, glial, and skeletal muscle; Loayza-Puch et al. 2013; Rooijers et al. 2013; Gonzalez et al. 2014; Michel et al. 2018) and we assess potential functional enrichment using a GO term analysis (Ashburner et al. 2000). Finally, we assess the role for SD in facilitating the formation of these TDGFs (Khurana et al. 2010).

## Materials and Methods

### Data Set Assembly and SSNs

Protein*-*coding DNA genes were downloaded from the Ensembl Genome Browser API (Version 71) (Flicek et al. 2014) for the following species (and versions): *Homo sapiens* (GRCh37), *Mus musculus* (GRCm38), *Pan troglodytes* (CHIMP2.1.4), *Gorilla gorilla* (gorGor3.1), *Macaca mulatta* (MMUL_1), *Pongo abelii* (PPYG2), and *Callithrix jacchus* (C_jacchus3.2.1) (supplementary table S1, Supplementary Material online). Sequence quality was assessed to ensure the coding sequences had complete codons, and any coding sequence containing intermittent stop codons indicative of sequencing error were removed. Coding sequences were then translated considering the phase information of each sequence, and a corresponding amino acid database was generated. A best reciprocal BLASTp (Altschul et al. 1990) analysis was carried out with e-value = 1×10^*−*^^5^ and self-hits were removed. A comparison of methods to detect gene fusions using SSNs (supplementary fig. S1, Supplementary Material online) was performed and MosaicFinder (Jachiet et al. 2013) was chosen as it was the most conservative. MosaicFinder deconstructs global SSNs into discrete subgraphs and employs mathematical graph decomposition to identify clique minimal separators (gene fusions). To accommodate different rates of change, three thresholds of sequence identity (SI) (70%, 80%, and 90%) were used in MosaicFinder (Jachiet et al. 2013). iGraph was used to visually inspect each fusion/parent gene family. Protein-coding sequences for gene families associated with each gene fusion event were extracted from our database. Alignments were constructed using PRANK (Loytynoja and Goldman 2005) for each fused gene and all corresponding parent genes. False positives that occur due to distant homology of parent genes were removed after careful manual inspection of all alignments (Edgar 2004).

In order to determine the phylogenetic distribution of the fused genes, an RNA data set was assembled that spanned the vertebrate phylogeny. The RNA data sets used were taken from the NCBI database (Sayers et al. 2009) for the following: bonobo; cat (Felis_Catus_3.2); coelocanth (LatCha1); chicken (Gallus_gallus4.0); chimp (PanTro4); cow (BosTau4); dog (CanFam3.1); dolphin (Ttru_1.4); elephant (Loxafr3.0); fugu (FUGU4.0); gibbon (Nleu_1.0); gorilla (Gorgor3.1); guinea pig (Cavpor3.0); horse (EquCab2.0); human (GRCm38.p3); macaque (Mmul_051212); marmoset (Callithrix_jacchus3.2); brown bat (MyoLuc2.0); mouse (GRCm38.p2); naked mole rat (hetGla2/hetGla_Female_1.0); olive baboon (Panu2.0); opossum (MonDom5); orangutan (P_pygmaeus2.0.2); orca (Oorc1.1); pig (Sscofra10.2); platypus (Ornithorynchus_anaticus5.01); rat (Rnor.6); tarsier (Tarsius_syrichta1); turkey (Turkey2.01); zebrafish (GRCz10), and zebrafinch (teaGut3.2.4). Sequence similarity searches were performed using the fused genes as queries (Altschul et al. 1990). Results were parsed and alignments generated using MUSCLE (Edgar 2004). (Note: in this instance, MUSCLE [Edgar 2004] is used rather than PRANK [Loytynoja and Goldman 2005] as it had adequate sensitivity and increased speed). A functional enrichment analysis was carried out using the software package GOrilla (Eden et al. 2009), the Ensembl gene identifiers (Flicek et al. 2014) for fused genes and their parents from human and mouse at each SI threshold (70%, 80%, and 90%) were used. GOrilla calculates an exact *P* value and accounts for multiple testing through an FDR *q* value calculation. For comparative purposes, this was followed by a functional enrichment analysis using DAVID (Huang et al. 2007). GO terms for each fused gene were obtained (Ashburner et al. 2000) (supplementary table S2, Supplementary Material online).

### Analysis of Regions of SD

To assess the frequency of occurrence of fused genes and parent genes in regions of SD, simulations were carried out as follows: Human chromosomal positions were obtained for all fused genes and their parents from Ensembl (Version 74) (Flicek et al. 2014). SD coordinates for the human genome were taken from the Segmental Duplication database (She et al. 2006). Overlap between human fused/parent gene chromosomal coordinates and the human SD coordinates was assessed. The coordinates for all human protein*-*coding sequences were downloaded from the Ensembl Genome Browser (Version 74) (Flicek et al. 2014). Randomly sampled data sets of fused and parent genes were generated. This was done by generating data sets of 37 genes in size by random sampling from the entire set of protein*-*coding genes without any restriction on chromosomal location. For each randomly sampled data set, the number of genes that located to regions of SD were recorded. This simulation was carried out on 10,000 replicate sets and *P* values were obtained.

### Gene Expression Analysis from Previously Published RNA Sequence Data Set

To determine the level of expression of the unique breakpoints of the fusion genes, we used previously published RNAseq data sets as follows: Illumina Genome Analyser IIx sequence reads were downloaded from the SRA archive on the NCBI browser, project number SRP007412 (Brawand et al. 2011). This data set was chosen as at the time of analysis it represented the highest quality transcript sequencing information from six primates from a range of six tissues. Reads were predominantly 76 base pair single-end sequences (paired-end sequences were discarded due to poor quality). Sequences were downloaded for all seven species in the data set (i.e., human, chimpanzee, macaque, marmoset, gorilla, orangutan, and mouse), and for all six tissues (i.e., brain, cerebellum, kidney, heart, liver, and testis) (Brawand et al. 2011). SRA files were converted to SAM format using the SRA toolkit (Leinonen et al. 2011) and then to FASTQ format using SAMtools (Li et al. 2009). Reads were quality checked using FASTqc (Patel and Jain 2012). The following characteristics of sequence reads were determined per base: sequence quality, quality scores, sequence content, GC-content, N content, and per sequence for GC-content, length distribution, overrepresented sequences, and kmer content. Phred scores were low for all reads because of the IBIS base caller had been used in the initial study (Kircher et al. 2009). Reads with phred scores <20 were removed. The leading 10–13 bases of each sequence read were also of poor quality (supplementary fig. S2, Supplementary Material online), possibly due to presence of adaptor sequences, and they were trimmed using TrimGalore (v0.3.3) (http://www.bioinformatics.babraham.ac.uk/projects/). Finally, reads were again inspected by FASTqc.

Reference genomes for human, chimpanzee, macaque, marmoset, orangutan, and mouse were downloaded from the Ensembl Genome Browser (Version 74) (Flicek et al. 2014). The filtered reads for each species were mapped onto the corresponding reference genome using STAR (Dobin et al. 2013). In the case of fused genes, only reads that span the junction/breakpoint of both parents were mapped (supplementary fig. S3, Supplementary Material online). Reads that mapped successfully were then counted on a species-by-species basis. For each species, the genome annotation file (“.gtf”) was downloaded from the Ensembl Genome Browser (Flicek et al. 2014). HTseq Count software package (Version 0.5.3p3; http://www-huber.embl.de/users/anders.HTSeq) was used to identify the reads that mapped to annotated transcripts and to count the number of reads mapped per transcript (the union overlap resolution method was used to deal with overlapping sequences). Transcripts containing >1 mapped read were considered to be expressed; however, analyses were also carried out at >3, and at >5, mapped reads (supplementary file 5, Supplementary Material online). As expected, and across all species examined, the most stringent threshold of >5 reads resulted in the least number of reads mapping to fusion breakpoints and using the most lenient threshold of >1 yielded the largest number of confirmed fusion breakpoints. As we were only mapping across the 50-bp fusion breakpoint—the number of reads that would map to this small region were already limited. In addition, “new” genes are generally thought to have a lower expression level. Therefore, we present the results from the >1 category as evidence that this region is transcribed and not the result of an annotation error. Fused genes identified at 90% identity threshold were then assessed for expression patterns.

As justified earlier, we considered a fused gene to be “expressed” (in a given species and tissue) when the region spanning the junction of the fused gene was mapped by at least one read. Reads that mapped to fused gene families at each percentage identity (70%, 80%, and 90%) were extracted. In this way, we calculated the percentage of fused genes and parent genes expressed in each species and each tissue. To test whether there were significantly more fused gene families expressed in a particular tissue in comparison to other tissues, we calculated the *Z*-score, one tailed, and two tailed *P* values. An analysis of the TPM (transcript per million) values for fusion breakpoints as compared with the rest of the transcriptome, confirms that the rates of mapping to the fusion gene breakpoints is higher than background mapping rates (supplementary file 5, Supplementary Material online).

### Mapping Fused Genes in the Context of Phylogeny

Using the fused genes for which we had evidence of transcription, we blasted other available reference transcriptomes in order to determine whether these breakpoints were transcribed in other species outside of the great apes and/or human lineages. Fused gene sequences identified were obtained from the Ensembl Genome Browser (Flicek et al. 2014) (Version 73) and pairwise alignments against each individual parent were prepared using MUSCLE (Edgar 2004) in order to obtain breakpoint locations. “Fusion breakpoint” reads were constructed by cleaving each fused gene sequence, incorporating only the region spanning the fusion junction (50 bp both sides of fusion breakpoint). RNA sequence reads of Opossum, Lizard, Putterfish, Frog, and Chicken (Brawand et al. 2011) were then mapped onto their corresponding reference genomes (Flicek et al. 2014). BlastN (Altschul et al. 1990) was used to search the RNA sequence reads for matches to the “fusion breakpoint” read (supplementary fig. S3, Supplementary Material online). BlastN allows more mismatches than other local alignment tools—a property that is preferable in this case due to divergence times between the species under consideration.

### Gene Expression Analysis

Htseq count results were used to carry out a differential gene expression analysis using the EdgeR package in R (Robinson et al. 2010). Here, both fusion and parent gene expressions were investigated for each tissue sample within each species.

### Qualitative RT-PCR

To complement the RNAseq data analyses, we carried out RT-qPCR analyses to investigate expression of the unique fused gene breakpoints in a range of tissues. Total human RNA was purchased from Life Technologies and RNA was extracted from the following tissues: liver (AM7960), brain (AM7962), placenta (AM7950), lung (AM7968), and testes (AM7972). About 5* *µg was digested with DNAseI (Sigma AMP-D1) for 15 min at room temperature (RT). cDNA was synthesized from the DNAse-free RNA using the Tetro cDNA synthesis kit (Bioline BIO-65042) as per manufacturer’s instructions. Quantitative real-time PCR was carried out on the cDNA using ABI fast SYBR-green qPCR kit (4385616) and on the 7900 HT ABI thermal-cycler. Each reaction contained 20 ng/µl cDNA amplified with 0.2 µM of each primer, this was carried out in triplicate. Primer sequences and their targets can be found in supplementary file 4, Supplementary Material online, and ACTB was used as an internal reference. Expression was assessed in two ways: 1) The primer pair displayed a single reproducible dissociation curve in at least one tissue analyzed, and 2) The delta CT value for a given primer pair compared with ACTB >0.1, which we determined was our detection limit of a true positive.

### Ribosome Profiling Data Analysis

To determine whether there is evidence for translation of these fused genes from existing ribosome profiling data, we carried out the following analysis: Human ribosomal profiling data sets were selected from the GWIPS Web Browser (Michel et al. 2018). SRA files were downloaded (Leinonen et al. 2011) (GSE45833, Loayza-Puch et al. 2013; GSE51424, Gonzalez et al. 2014; GSE48933, Rooijers et al. 2013; GSE56148, Wein et al. 2014). These data sets were selected as they were the most recent high*-*quality ribosomal profiling data sets available. FASTq file conversions were carried out using fastq-dump package from the SRAtoolkit (Leinonen et al. 2011). Adaptors were removed and reads were trimmed using the Fastx-toolkit’s (http://hannonlab.cshl.edu/fastx_toolkit/index.html*)* fastx_trimmer function and cutadapt (Martin 2011), and reads of >25 nucleotides were retained (supplementary fig. S2, Supplementary Material online). Data quality was assessed using the FASTQC package (Andrews 2015) after each cleaning step. rRNA depletion of each data set was carried out using BowTie2 (Langmead and Salzberg 2012) against a human rRNA data set (Quast et al. 2013). About 16 bp fusion gene reads were constructed, each read spanning the fusion breakpoint equally. Reads were mapped to each cleaned ribosequence data set using the Bowtie2 (Langmead and Salzberg 2012) function to allow for split read mapping. Reads hitting each data set where then further mapped to the latest human RefSeq genome (Hg19) (O’Leary et al. 2016) available on the UCSC Genome Browser (Tyner et al. 2017) again using the BowTie software package (Langmead and Salzberg 2012) in order to obtain the chromosomal coordinates of each positive read hit. Positive hits were also confirmed visually on the IGV Web Browser (Robinson et al. 2011).

### Transcriptional Motif Enrichment

To investigate if there were specific transcription factor binding sites (TFBSs) associated with fused genes, we carried out an analysis of the regions around the transcription-mediated fusion genes using the JASPER CORE data set (Mathelier et al. 2016). The JASPER CORE data set consists of experimentally validated and manually curated TFBS across eukaryotic species. TFBS analyses were carried out using JASPAR’s profile inference package which firstly calculates a position frequency matrix for the TFBS of its corresponding TF and from this a position weight matrix can be calculated for each TF located within each input sequence (Stormo 2013). The calculation of each position weight matrix is based on an additive probabilistic model which assumes independence between nucleotides in the TFBS sequence motif (Stormo 2013). This analysis is complemented by a transcription factor flexible motif (TFFM) analyses which does not assume nucleotide independence but rather uses HMMs to calculate dinucleotide dependences and length flexibility of each TFBS (Stormo 2013). This algorithm predicted a panel of TFBS for each TDGF. The frequency of each TFBS was summed and from this the most a barplot constructed to highlight the most prominent TFBS per gene fusion (Stormo 2013). The expression profile of the TF corresponding to each TFBS was assessed using the Expression Atlas’ ENCODE data set (Kapushesky et al. 2010), this was to identify any potential TF driven expression profile of TDGFs across human tissues.

### Splice Factor Binding Sites across Fusion Genes Using Sfmap

To predict potential SFs across TDGFs, the Sfmap software package (Paz et al. 2010) was used. The Sfmap data set consists of known SF binding sites (SFBS). The frequency of each SFBS predicted with a score >90 was calculated across each fusion gene. The expression profile of each SF was analyzed using Expression Atlas’ ENCODE data set (Kapushesky et al. 2010) to assess SF over/under expression across human tissues. An additional, more specific, SF analysis was carried out on the fusion breakpoint sequence of each TDGF. Fasta formatted sequences of the intron and two exons (one from each parent) where the fusion occurred were downloaded from the Ensembl Genome Browser (Version 90) (Aken et al. 2017). Results were analyzed and interpreted in the same fashion as per previous SFmap experiment.

### Epigenomic Marker Analysis Using 127-Epigenomes

To determine whether the histone markers present in the fused genes corroborate the transcriptional profiles we observe from RNAseq and RT-qPCR analyses, we carried out an analysis of the epigenomic profile of these regions. Epigenomic profile data sets across a panel of human tissues were selected for five of the following histone markers: H3k27me3, H3k36me3, H3k9ac, H3k4me1, and H3k4me3 (Bernstein et al. 2010). These five markers were selected as they had the most data available across the broadest number of human tissues, as well as being associated with both transcriptional activation (e.g., H3k36me3) and repression (e.g., H3k9me3). Histone markers in TDGFs across the following epigenomic data sets were assessed: H3k36me3, GSM409312, GSM428296, GSM433176, GSM450268, GSM1013143, GSM956014, GSM906402, GSM669982, GSM910570, for H3k9ac GSM410807, GSM433171, GSM434785, GSM537705, GSM670021, GSM772811, for H3k4me1, GSM409307, GSM433177, GSM466739, GSM1013148, GSM1127129, GSM537706, GSM670015, GSM610025, GSM773001, GSM910575, GSM910576, for H3k4me3 GSM409308, GSM410808, GSM433170, GSM469970, GSM537967, GSM773005, GSM910561, GSM915336, and h3k27me3, GSM428295, GSM433167, GSM434776, GSM537698, GSM772833, GSM908952, GSM910563, and GSM112713 (Bernstein et al. 2010). These data sets contain epigenomic profiles from human tissues spanning embryonic stem cells, liver, brain frontal lobe, heart, placenta, kidney, ovary, lung, and pancreas. In*-*house software was used to obtain the subset of epigenomic data for transcription-derived fusion coordinates (obtained by Ensembl Genome Browser; Aken et al. 2017). The frequency of each marker across each tissue per gene was then analyzed and individual barplots constructed.

After this epigenetic profiles of all activation (e.g., transcriptional start sites and enhancers) and repressive (e.g., heterochromatin regions and repressive polycombs) motifs were assessed across 127 epigenomes (Bernstein et al. 2010). These data were based on a 15-state chromatin model implemented on 127 epigenomes available from the Roadmap Epigenomics Browser (Bernstein et al. 2010). The frequency of each motif was assessed in order to investigate transcriptional activation/repression across TDGF sequences.

### Motif Enrichment Analysis

Fused genes identified at the 90% similarity threshold were investigated for regulatory motif enrichment using the AME function in the MEME software suite (Bailey et al. 2009). Transcripts were obtained using Ensembl Biomart (Version 83) (Herrero et al. 2016). Default settings were used with a threshold of significance of *P* < 0.05 and shuffled input sequences were used as controls. Fused gene sequences were analyzed against a eukaryote DNA database (Herrero et al. 2016).

### Branch Length Estimation

We wished to determine whether there is a significant difference in the rate of change in fusion genes in comparison to nonfused. The branch length for each fused gene was estimated using the heterogeneous phylogenetic modeling approach implemented in P4 (Foster 2004). We estimated the branch lengths for all 24 alignments (12 fused genes each with 2 parents). For each estimate, we supplied P4 with an alignment and its associated precalculated composition vector and exchange rate matrix (e.g., JTT), and a fixed topology (species tree) (Thomson and Shaffer 2010; Morgan et al. 2013; Tarver et al. 2016). P4 was run for two million generations with sampling every ten generations. Parameters were assessed during the MCMCMC process and were accepted between 10% and 80% of the time. Finally, we compared the standard deviation between the checkpoints of the MCMCMC process, where a low standard deviation between checkpoints indicates convergence. To test if the model (composition vector and exchange rate matrix) used on each alignment was appropriate for the data, we carried out posterior predictive simulations. The simulations were generated during the MCMCMC process for each alignment. Each simulated data set was compared with the input data. The real data should look characteristically similar to the simulated data in instances where the model of evolution is adequate for the given data. This simulated data were then compared with the real data using a χ^2^ test to determine whether the fused genes were evolving at a faster rate on an average. For each analysis, *P* values were calculated based on the degrees of freedom for that analysis.

## Results

### TDGFs Are Detectable Using Graph Theory and RNAseq Data

**Fig. 1.**
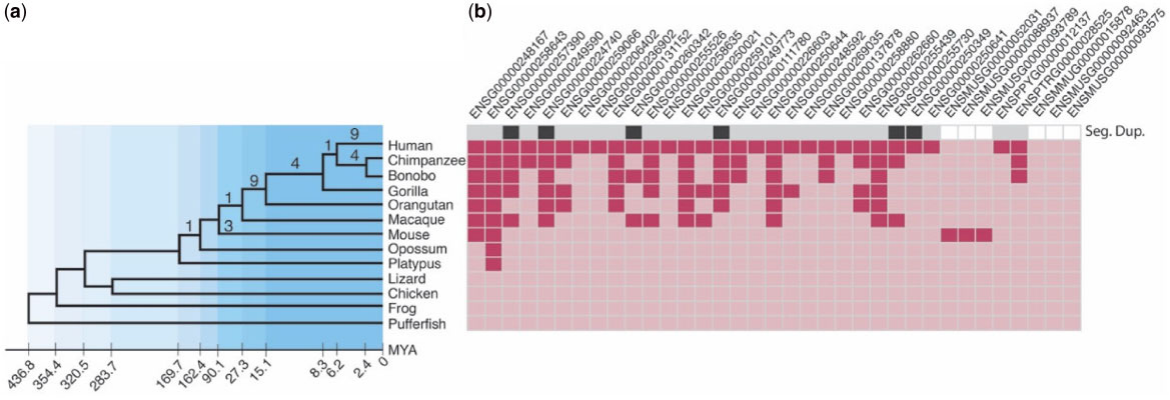
—Phylogenetic distribution of transcription-derived gene fusions (TDGFs). (*a*) The species sampled are represented in the phylogeny on the left with their estimated divergence times—Mya. Numbers on branches represent the number of gene fusions at those nodes. (*b*) Deep and pale pink cells in the matrix on the right correspond to the presence (deep pink) or absence (pale pink) of the gene fusion in that species. The “Seg Dup” row in the matrix shows the fused genes present at known segmental duplication breakpoints from human (dark gray), in pale gray are gene fusions for which there is missing information and in white are the gene fusions that are not found in human.

Protein SSNs (supplementary fig. S1, Supplementary Material online) were created using a best reciprocal BLAST (Altschul et al. 1990) search of human, five nonhuman primates, and mouse (supplementary table S1, Supplementary Material online). The sequence similarity searches were performed at three levels of SI between parent and fused gene: 90%, 80%, and 70%, where the percentage value refers to the level of shared SI between the parent gene and the corresponding region of the fused gene (supplementary fig. S1, Supplementary Material online). The results for the 90% SI threshold are described here (for results for 80% and 70% SI thresholds see supplementary file 1, Supplementary Material online). Fused genes detected at 90% SI were compared with seven nonprimate vertebrates (mouse, opossum, platypus, lizard, chicken, frog, and fugu) using RNAseq data (Brawand et al. 2011; Coordinators 2016) allowing us to place the origin of fused genes more precisely in on the phylogenetic tree (fig. 1).

### TDGFs Can Be Lineage-Specific and Can Evolve Alternative Splice forms

Using SSNs, we identified a total of 45 fused genes across our data set (Human, Chimp, Gorilla, Orangutan, Macaque, and Marmoset and Mouse) using the 90% SI threshold (unsurprisingly 80% and 70% SI thresholds yielded a greater number of fused genes—68 and 98, respectively) (supplementary file 1, Supplementary Material online). To place each fused gene in a phylogenetic context and to investigate their RNA expression profiles, we searched the fused genes against high*-*quality transcriptome data for human, chimp, bonobo, gorilla, orangutan, mouse, fugu, frog, and lizard (Brawand et al. 2011; Coordinators 2016) (fig. 1). In total, 35 TDGFs could be tested using available RNAseq data and 32 of these produce RNA transcripts (Brawand et al. 2011), three of which only have transcripts in mouse. Nine TDGFs have subsequently evolved annotated alternatively spliced transcripts in human (Herrero et al. 2016). Interestingly, four of the nine human-specific genes and all three of the mouse-specific genes have annotated alternative transcripts (Herrero et al. 2016) (supplementary file 2, Supplementary Material online). To test if the evolutionary rate of fused gene families was different across the great apes—branch lengths were compared. We found no significant difference in branch lengths of TDGFs across species suggesting that TDGFs are evolving at similar rates across the Great Apes.

### TDGFs Are Enriched for Specific Functions

An analysis of the function of parent genes using GOrilla (Eden et al. 2009) reveals they are functionally biased. Sufficient power exists for a statistical test of the fusion genes from the 70% SI (fused genes = 98, parent genes = 1,615) and 80% SI (fused genes = 68, parent genes = 417) set (supplementary file 3, Supplementary Material online). The results indicate that the parent genes showed enrichment for DNA binding (70% SI: *P* value = 7.41e-37, FDR = 2.32e-34), (80% SI: *P* value = 1.02E-16, FDR = 2.65E-14) and nucleic acid binding (70%: *P* value = 1.30e-31, FDR = 2.03e-31), (80%: *P* value = 3.20E-13, FDR = 4.16E-11) (supplementary table S2, Supplementary Material online). Interestingly, for TDGFs, there is a bias for enzymatic functions and mediation of protein interactions.

### Genomic Location of SDs and TDGFs Overlap

Of the 45 fused genes (90% SI), 26 have been mapped to specific loci in the human reference human genome (GRCh38) (Smedley et al. 2015) and 8 out of 26 map to known regions of SD (She et al. 2006) (fig. 1). To investigate whether the co-occurrence of fused genes and SD breakpoints was significantly higher than expected, we randomly sampled protein-coding gene sets of the same size (i.e., 26 genes) 10,000 times, and assessed their frequency of co-occurrence with SD breakpoints and compared results. If SD drives gene fusion, we would expect to see gene fusions localizing to SDs. Indeed, we find fused genes are significantly more likely to occur at known SD regions (*P* value = 0.0282). Though 26 genes is a small sample size, taken together, these results suggest a role for SD in the emergence of new genes by TDGF.

### TDGFs Are Not Tissue-Specific in Expression

**Fig. 2.**
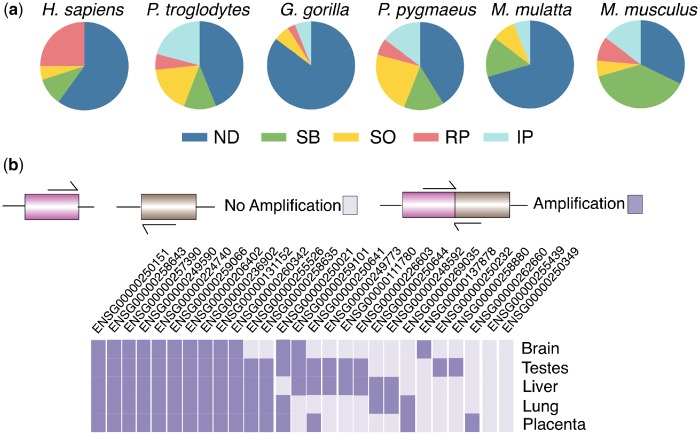
—Expression profiles for transcription-derived gene fusions (TDGFs) and their parent genes. (*a*) Comparison of the expression profiles between the orthologs of the human-specific fusion genes and their respective orthologous parent gene counterparts in each vertebrate shown. RNAseq data (Brawand et al. 2011) of each organism from the cerebellum, brain, heart, kidney, liver, and testis* (*not available for *Pan troglodytes* and *Macaca mulatta* data sets) were analyzed for the presence of >1 read that maps the breakpoint for each gene fusion. Sample sizes were as follows: *Homo sapiens* (20); *P. troglodytes* (34); *Gorilla gorilla* (34); *P. pygmaeus* (34), *M. mulatta* (34), and *Mus musculus* (34). ND, no expression detected; SB, same expression as both parent genes; SO, same expression profile as one parent gene; RP, reduced breadth of expression compared with parent genes; IP, increased breadth of expression compared with parent genes. (*b*) RT-qPCR to determine the expression of each fused gene across a panel of five human tissues. Darker cells represent amplified product and presence of the gene fusion in that human tissue, pale squares represent no evidence for the gene fusion transcript in that tissue.

To determine the range of human tissues where the 45 fused genes are expressed, we analyze RNAseq data for seven species: human, chimpanzee, gorilla, orangutan, macaque, marmoset, and mouse (Brawand et al. 2011) (fig. 2*a*). The RNA expression of fused genes is determined from the RNAseq raw reads that map specifically to the fusion breakpoint. Expression of the fused genes across all seven species is compared with the average gene expression in liver, heart, cerebellum, kidney, and testis, and we find no significant enrichment of fused gene expression in any single tissue (supplementary table S3, Supplementary Material online)*.* However, on analyzing the expression on a species-by-species basis, we find elevated numbers of fused genes expressed in the brain, liver, and heart in four species (supplementary file 2, Supplementary Material online).

Previous analysis of expression patterns of 1:1 protein-coding orthologs (Brawand et al. 2011) revealed, perhaps unsurprisingly, that brain and cerebellum share a more similar expression profile than either does with liver, kidney, testes, or heart tissues in all seven species (Brawand et al. 2011). Although brain and cerebellum are more similar when compared with other tissues, comparative transcriptome studies have shown differential gene expression patterns between these two tissues (Chen et al. 2016). We find between one and seven fused genes have signatures of DE between cerebellum and brain across the five Great Apes tested (Human, Chimp, Gorilla, Gibbon, and Macaque) (supplementary table S3, Supplementary Material online). Intriguingly, out of the seven fused genes in human, DE is manifest between the following tissues (number of fused genes in parentheses): brain* (*includes cortex and temporal lobe) and cerebellum (3); brain and testes (5); between brain and heart (2); brain and kidney (1), and brain and liver (1). Therefore, although 1:1 orthologs generally tend not to have DE between brain and cerebellum, the human fused genes do display DE patterns between these tissues, highlighting variation in expression of these new fused genes.

To precisely assess RNA expression of the TDGFs, we undertook RT-qPCR on the breakpoint of suitable fusion transcripts in the following five human tissues: testis, liver, lung, brain, and placenta (table 1 and supplementary file 2, Supplementary Material online). TDGF suitability for this test was judged based on the ability to generate unique primers that span the fusion breakpoint for each fusion transcript—26 out of 33 human transcripts met this criterion. The RNA expression of 24/26 fused transcripts in these human tissues can be confirmed (fig. 2*b*). Similar to the findings from our RNAseq metadata analysis (Brawand et al. 2011), we see no distinct tissue-specific expression pattern for fused transcripts: three transcripts are expressed in a single tissue, whereas ten fused transcripts are expressed in all five tissues. In total, 13 fused transcripts are expressed in brain, 19 in testes, 17 in placenta, 19 in liver, and, 16 in lung (fig. 2*b*). Therefore, unlike other new genes the expression of transcription-mediated fused genes is not confined to a single tissue—and certainly not just to the testis although testis is usually represented as one of the tissues in which expression is detected.

**Table 1:** Results of RT-qPCR on 26 TDGFs in 5 human tissues

Tissue	Number of Fusions Expressed
Brain	13
Testis	19
Liver	19
Placenta	17
Lung	16

Out of the 26 testable TDGFs, we display the number that are detected as expressed following RT-qPCR in each of the five human tissues assessed.

### TDGFs Have Evidence of Translation from Ribosome Profiling Data

**Fig. 3.**
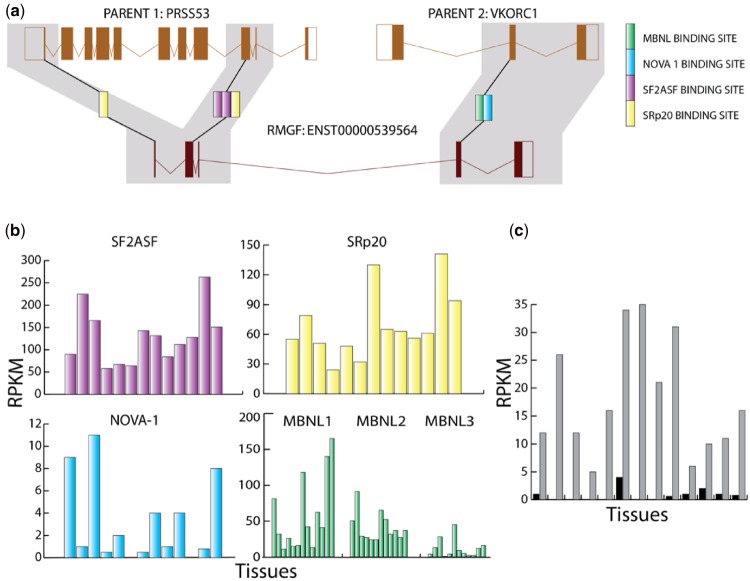
—Splice Factor Binding site profiles for fusion transcript ENST00000529564 and the corresponding parent genes. (*a*) Transcription-derived gene fusion transcript ENST00000529564 is displayed along with parent genes *PRSS53* and *VKORC1*. Splice Factor binding sites for splice factor “SF2ASF” (in pink), “MBNL1-3” (in gray), “SFp20” (in red), and “NOVA1” (in blue). Each square represents a single SFBS present. (*b*) Expression level of each Splice factor binding site across ENST00000529564 across a panel of tissues on the *x* axis (left to right): Adipose tissue; Adrenal gland; Brain; Heart; Kidney; Liver; Lung; Ovary; Pancreas; Sigmoid colon; Small intestine; Spleen, and Testis. Expression data are given in RPKMs. Expression data were obtained from the expression atlas ENCODE data set (Kapushesky et al. 2010). (*c*) Expression profile of Splice factor binding sites of each of the parent genes *PRSS53* (gray bars) and *VKORC1* (black bars). Tissue panel on the *x* axis (left to right): Adipose tissue; Adrenal gland; Brain; Heart; Kidney; Liver; Lung; Ovary; Pancreas; Sigmoid colon; Small intestine; Spleen, and Testis. Expression data are given in RPKMs. Expression data were obtained from the expression atlas ENCODE data set (Kapushesky et al. 2010).

Subsequently, to investigate the translation of novel RNA products (Ingolia et al. 2009; Aspden et al. 2014), we assessed the translatomic profiles of fusion transcripts across fibroblast, skeletal muscle, and glioma ribosome profiling data sets (Loayza-Puch et al. 2013; Rooijers et al. 2013; Gonzalez et al. 2014; Wein et al. 2014). In total, there were 19 fused genes out of the 45 that had unique sequence spanning the breakpoint of the fusion, and in total 3 fusion genes had ribosome footprints in fibroblasts (2 of these were expressed in all tissues from qRT-PCR analysis). Features of these three TDGFs with evidence of translation have been summarized in table 2. Expression of TDFG *ENST00000446072* was detected in human testes and liver tissues from RNAseq data analysis (Brawand et al. 2011) and across all tissues in our RT-qPCR (fig. 2). A single NOVA1 SF binding site was found to be located in the intron spanning the fusion breakpoint which may suggest increased expression in human (supplementary fig. S4, Supplementary Material online) (Ule et al. 2005). The expression of TDFG *ENST00000567078* (supplementary fig. S5, Supplementary Material online) is ubiquitous and the SF analysis again identified a NOVA1 domain within intron 2 (supplementary fig. S5*b*, Supplementary Material online) (Paz et al. 2010). Predominant HMGI/Y transcription factor use is also predicted for this TDGF which is indicative of an activated gene. We did not detect expression of TDFG *ENST00000529564* using RT-qPCR; however, the SF and TFBS predictions indicate a broad expression pattern as does the analyses of 127 epigenomes (Bernstein et al. 2010) (fig. 3).

**Table 2:** Splice factor and transcription factor binding sites predicted for 3 of the TDGFs

Transcript_ID	RT-qPCR	Predicted Parents	SFBS	TFBS
ENSG00000446072	Ubiquitious	N/A	NOVA1	N/A
ENSG00000567078	Ubiquitious	ARL6IP1 and RPS15A	NOVA1	HMGI/Y
ENSG00000529564	No expression	PRSS53-201 and VKORC1-206	SFASF, SRp20, mbnl, NOVA1	Sp1, Zfx, YGR067C

only those transcription derived gene fusions for which we had evidence of translation from ribosome profiling datasets were used in this analysis

## Discussion

Regions prone to nonallelic homologous recombination in genomes have shown that they are enriched with transcripts particularly in primate species. Nonallelic homologous recombination can be caused by clustered repeated sequences, such as SDs. The range of duplicated blocks varies from species to species; however, some general trends have been described, for example, mice contain less SDs in comparison to tandem duplications, whereas the converse is true in primates. It has previously been proposed that regions of SD may contain a high proportion of fusion transcripts (Marques-Bonet, Kidd, et al. 2009). Indeed, we observe that 8/26 of our TDGFs that we could map precisely are present at known SD breakpoints which provides empirical support for enrichment of fusions at SD breakpoints; however, our sample size is small. Investigations of ENCODE data have revealed that ∼4–5% of genes have the potential to generate readthrough transcripts of this nature (Nacu et al. 2011). Regardless of the overall number of TDGFs present, it is widely understood that they contribute to proteome diversity and regulatory functions.

Fusion genes that have previously been validated tend to be associated with receptor and enzymatic functions (Akiva et al. 2005). For example, CCL14/CCL15 is a chemokine receptor (Stone et al. 2017), CYP2C18/CYP2C19 is an enzyme involved in drug metabolism (Lofgren et al. 2008) and the SBLF-ALF fusion is a leutinizing hormone receptor (Xie et al. 2002). Our analysis of GO terms from the parents of the TDGFs in our data set revealed a bias toward binding activities (cation/ion, heterocyclic compounds, and nucleic acids) and endopeptidase activity but the small sample size of our TDGF data set make it difficult to draw comparisons about functional trends.

The TDGFs we identify in this study have the capacity to produce alternative transcript isoforms. In general, gene duplicates or members of large gene families tend to have a low number of alternative transcripts with similar expression profiles, while single copy genes are more likely to have a higher number of alternative transcripts with more heterogeneous tissue expression profiles. It has been shown that older gene duplicates tend to have more alternative transcripts than younger duplicates. These general trends may suggest that the number of alternative transcripts present for a given gene is an indicator of the length of time the gene has been in the genome (Iniguez and Hernandez 2017), and that TDGFs with multiple isoforms may have appeared earlier. However, the presence of multiple isoforms for TDGFs may be attributable to their location in the genome rather than age, that is, there may be a higher probability of transcriptional slippage in regions of genomic complexity such as in regions of SD (Ritz et al. 2011), and alternative transcripts across human protein*-*coding genes tend to not be shared among even closely related species (Iniguez and Hernandez 2017). Not all isoforms will produce protein products, indeed TDGFs *ENSG00000250151* and *ENSG000002500021* each have transcript isoforms that have been shown to regulate gene transcription through nonsense mediated decay (Reyes and Huber 2018).

In total, we determined differential gene expression patterns in three TDGFs in our data set. TDGF *ENSG000000137878* (or *GCOM1*) which is known to have multiple fused transcripts (processed and unprocessed) has differential expression across all tissues sampled. The processed transcripts are known to be involved in intracellular signal transduction in the nucleus while the unprocessed transcripts control the expression of *POLR2M* through nonsense-mediated decay (Roginski et al. 2004). TDGF *ENGS00000185304* (RANBP2-like and Grip domain-containing protein 2) has differential expression between brain and testes and between heart and cerebellum and is located in the nucleus. It plays a role in GTPase binding which has been shown to control nucleocytoplasmic transport, nuclear organization and both nuclear and spindle assemblies (Ciccarelli et al. 2005). Finally, TDGF *ENSG00000283154* (*IQJC-SCHIP1*) is differentially expressed in the brain in comparison to all other tissues examined and it is known to have a role in contributing to the maintenance of neuronal polarity through the Ca^2+^ and K^+^ channels found in the axon initial segment (Papandreou et al. 2015).

The open chromatin structure in testes, the increased expression of transcriptional machinery, and the selective pressures acting on the male germline all contribute to permissive transcription of new transcripts in the testes (Nyberg and Carthew 2017). Therefore, new genes are thought to be expressed initially solely in the testes and over time more broadly as described by the “out of testes hypothesis” (Marques et al. 2005; Vinckenbosch et al. 2006; Kaessmann et al. 2009; Kaessmann 2010). However, the TDGFs identified here have a broader expression signature most likely due to the fundamental nature of their formation from established genes and corresponding regulatory motifs. Our results indicate that TDGFs do not follow the same trend as would be expected of new genes that have emerged by other processes in the genome.

## Conclusion

Our network-based analysis of seven genomes has focused on a highly conservative subset, that is, PI of >90%. Due to sequence quality, divergence times and availability of alternative transcript data, the reported number of fused genes in nonhuman primates is most likely an underestimate. TDGFs are enriched in regions of human SD suggesting that the genomic instability typical of these regions aids in rearrangement of genes into neighborhoods that facilitate TDGF. Unlike other new genes, fused gene transcripts appear to have a broad RNA expression profile across tissues and cell types. We have provided evidence for the active translation into proteins for three of these TDGFs.

## Supplementary Material

Supplementary data are available at *Genome Biology and Evolution* online.

## Supplementary Material

evz163_Supplementary_Data
