# Supplementary material for: Gene Fusions Derived by Transcriptional Readthrough are Driven by Segmental Duplication in Human
Source: Genome Biol Evol. 2019 Aug 10;11(9):2678–90. doi: 10.1093/gbe/evz163 (PMC6764479; doi:10.1093/gbe/evz163)
Supplement: evz163_Supplementary_Data [file evz163_supplementary_data.zip › McCartneyetal_Supplementary_Information_June_2019.docx]

**Supplementary Tables**

**Table S1: Genome information for Species used in the study.**

| **Species** | **Genome_Build Identifier** | **Genome Size (Gb)** | **N50 of scaffolds (bytes)** | **N50 of contigs**  **(bytes)** | **Coverage** | **#protein coding genes** |
| --- | --- | --- | --- | --- | --- | --- |
| **Human** | GRCh37 | 3.2 | 44983201 | 38116554 |  | 20805 |
| **Chimpanzee** | CHIMP2.1.4 |  | 8925874 | 50656 | 6X | 18759 |
| **Gorilla** | GorGor3.1 | 3.04 | 913458 | 11657 | 35X | 20962 |
| **Orangutan** | PPYG.2 | 3.08 | 135000294 | 15654 |  | 20424 |
| **Macaque** | MMUL_1 | 3.01 | 24300000 | 2700 | 5.1X | 21905 |
| **Marmoset** | C_jacchus3.2.1 | 2.75 | 5167444 | 29273 |  | 20993 |
| **Mouse** | GRCm38 |  | 52589046 | 32273079 |  | 23148 |

***Table S1:*** *Details of the species used in the study, the versions of the genomes and a measure of genome quality/completeness are shown. Entries in grey are unknown values.*

**Table S2: Results of functional enrichment analysis (using GOrilla).**

| **GO Term** | **Species** | **% Identity** | **Fusion Relationship** | **Description** | **P-Values** | **FDR q-value** |
| --- | --- | --- | --- | --- | --- | --- |
| GO:0003677 | Human | 90 | Parent | DNA binding | 7.41E-37 | 2.32E-34 |
| GO:0003676 | Human | 90 | Parent | Nucleic acid binding | 1.30E-31 | 2.03E-29 |
| GO:1901363 | Human | 90 | Parent | Heterocyclic compound binding | 1.08E-26 | 1.13E-24 |
| GO:0097159 | Human | 90 | Parent | Organic cyclic compound binding | 1.08E-26 | 8.45E-25 |
| GO:0043169 | Human | 90 | Parent | Cation binding | 1.53E-24 | 9.57E-23 |
| GO:0046872 | Human | 90 | Parent | Metal ion binding | 1.53E-24 | 7.98E-23 |
| GO:0043167 | Human | 90 | Parent | Ion binding | 1.01E-20 | 4.52E-19 |
| GO:0005488 | Human | 90 | Parent | Binding | 1.27E-12 | 4.99E-11 |
| GO:0001071 | Human | 90 | Parent | Nucleic acid binding TF activity | 4.53E-08 | 1.58E-06 |
| GO:0003700 | Human | 90 | Parent | Sequence-specific DNA binding TF activity | 4.53E-08 | 1.42E-06 |
| GO:0003674 | Human | 90 | Parent | Molecular_function | 8.18E-07 | 2.33E-05 |
| GO:0005515 | Human | 90 | Parent | Protein binding | 1.47E-04 | 3.85E-03 |
| GO:0004175 | Human | 90 | Parent | Endopeptidase activity | 3.73E-04 | 8.98E-03 |

***Table S2:*** *Functional enrichment analysis using orthologs of parents and TDGFs in human. Only those terms with significant enrichment are shown. Significance taken at the P>>0.05 level.*

**Table S3: Differential Expression of TDGFs Identified Across 6 Primates and Mouse using RNAseq data.**


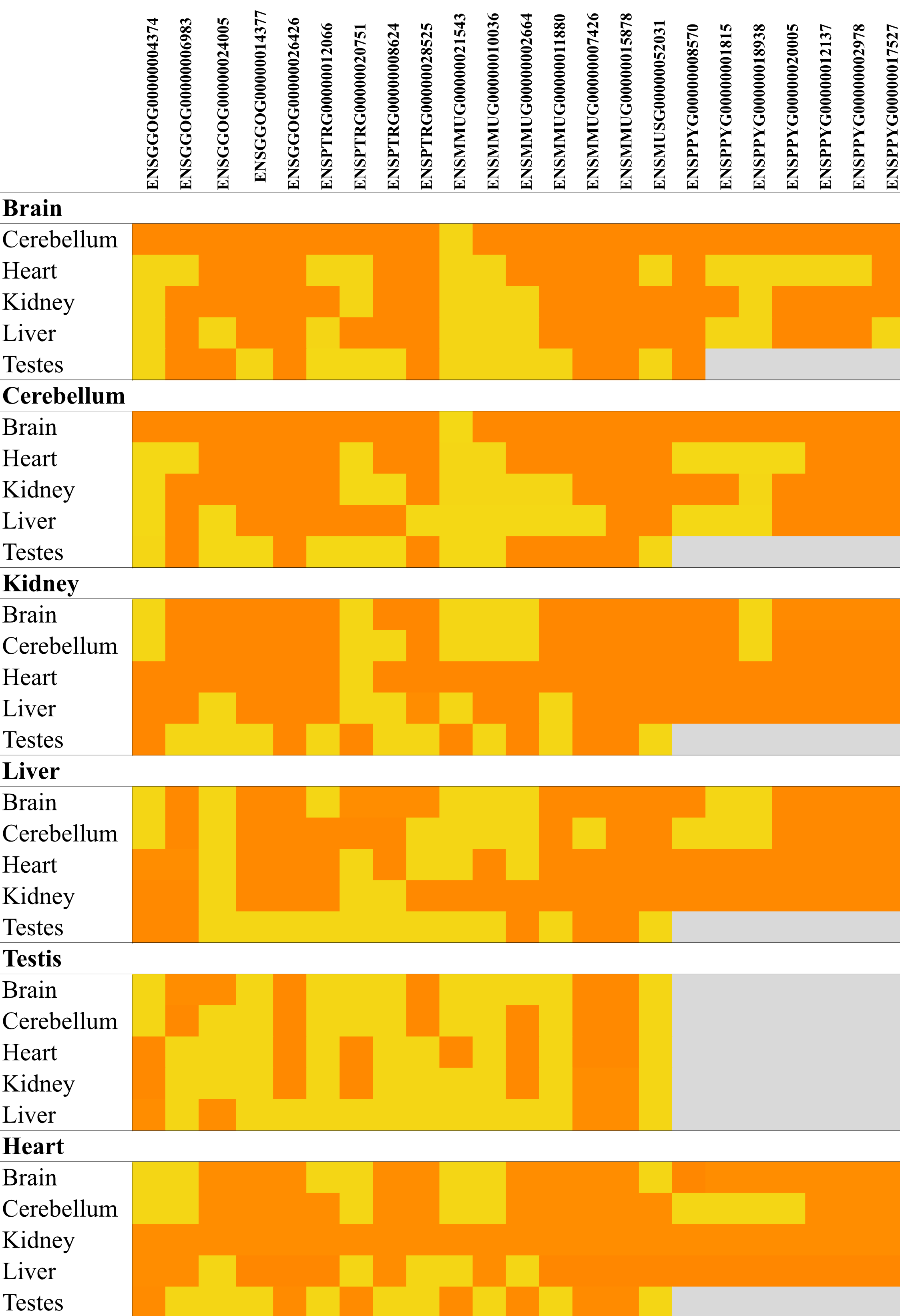


**Table S3:** *Metadata analysis of published RNA-seq data (Brawand et al, 2011).* *Comparison of differential expression patterns for 6 tissue types across 6 primate and mouse (Human, Gorilla, Chimp, Orangutan, Macaque and Marmoset). Yellow entries indicate no differential expression found between tissues. Orange entries represent differential expression between tissues. Grey entries indicate that data was unavailable to carry out the comparison.*

**Supplementary Figures**

**Figure S1 : Representation of transcription derived gene fusion and DNA-mediated gene fusion and their sequence similarity network relationship.**

**
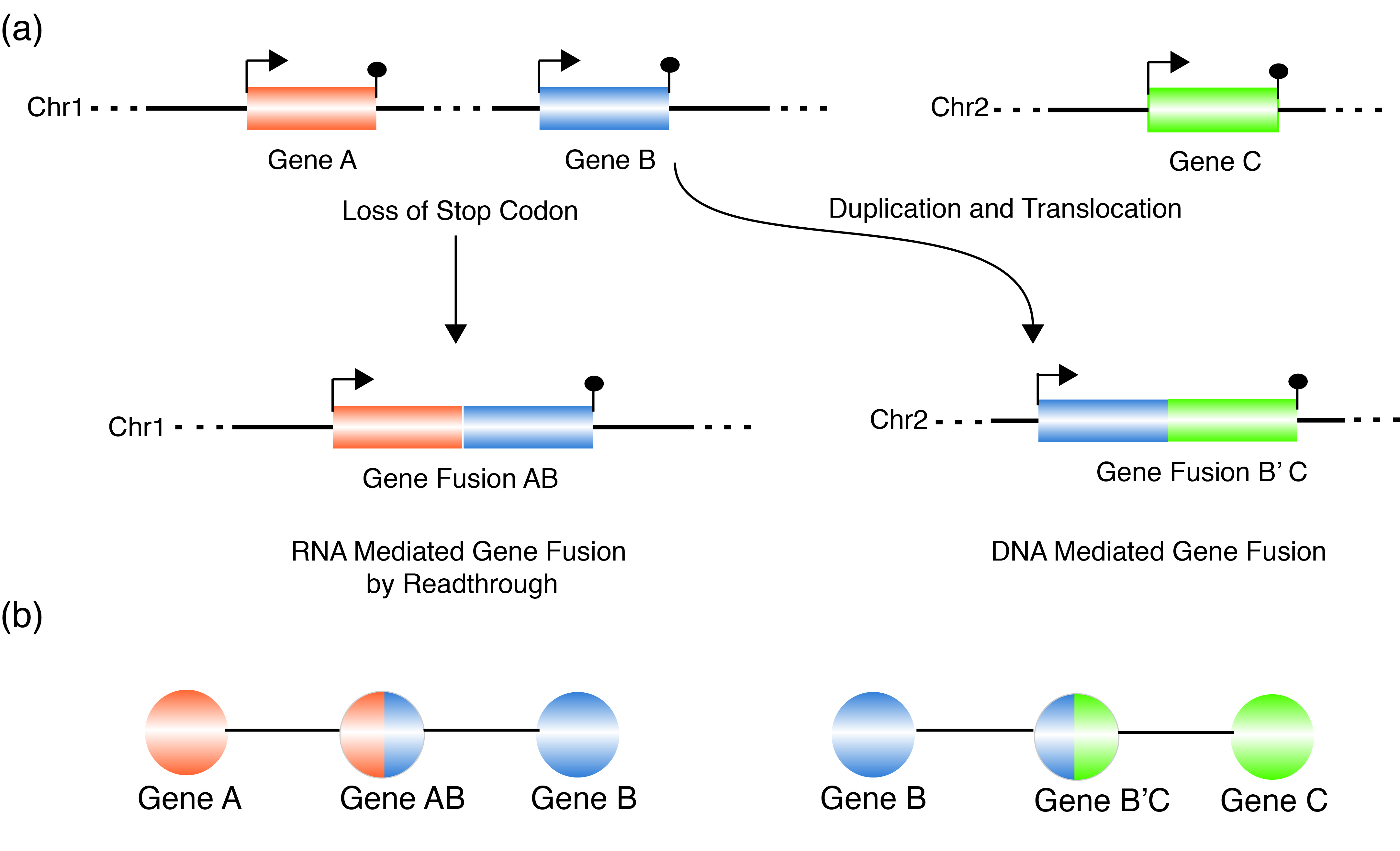
**

**Figure S1: *(a)*** *Two major mechanisms of gene fusion: Transcription derived gene fusion (TDGF) (left) and DNA-mediated gene fusion (DMGF) (right). TDGF: The loss, or ectopic splicing, of a stop codon leads to the generation of a fused transcript from the two adjacent parent genes A and B on Chr1. Alternatively, DMGF: duplication of Gene B to form B’ and the translocation of B’ to Chr2 and adjacent to gene C leads to fused gene B’C.* ***(b)*** *The “non-transitive triplet” relationships between the genes described in (a) and their fused genes as represented by a sequence similarity network. The circles represent the genes, the edges represent sequence similarity as determined through homology searches and the central node in each network represents the fused gene, e.g. Gene A and Gene B only share homology through their relationship through fused transcript AB and otherwise would be unconnected on the network.*

**Figure S2: Quality assessment of reads following editing by Trim_Galore.**

**
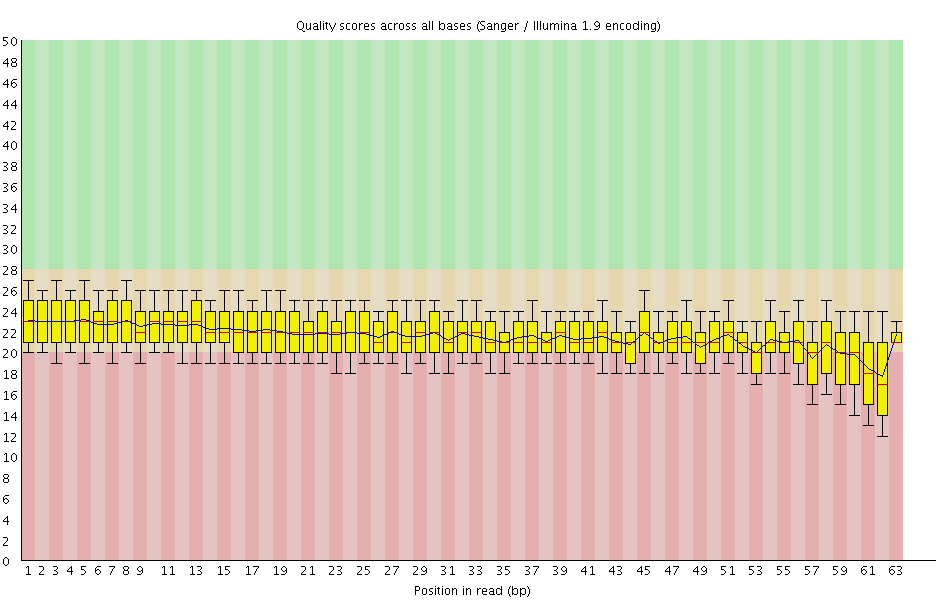
**

**Figure S2:** *FASTqc results of Human RNA sequence reads post trimming. Quality of reads after read removal (phred score of <20) and trimming the first 13bp of each sequence.*

**Figure S3**: **Approach used to map unique TDGF sequence reads**

**Figure S3:** *A “unique mapping” protocol was used where only those gene fusions containing unique reads (green strands) were retained as cases of TDGF. The green reads are unique to the RMGFs and share no homology with parent genes. Those reads that share homology with their parents (black strands) are not counted using this protocol*.

**Figure S4: Splice Factor (NOVA-1) Binding site profile for fusion transcript ENST00000446072.**

**
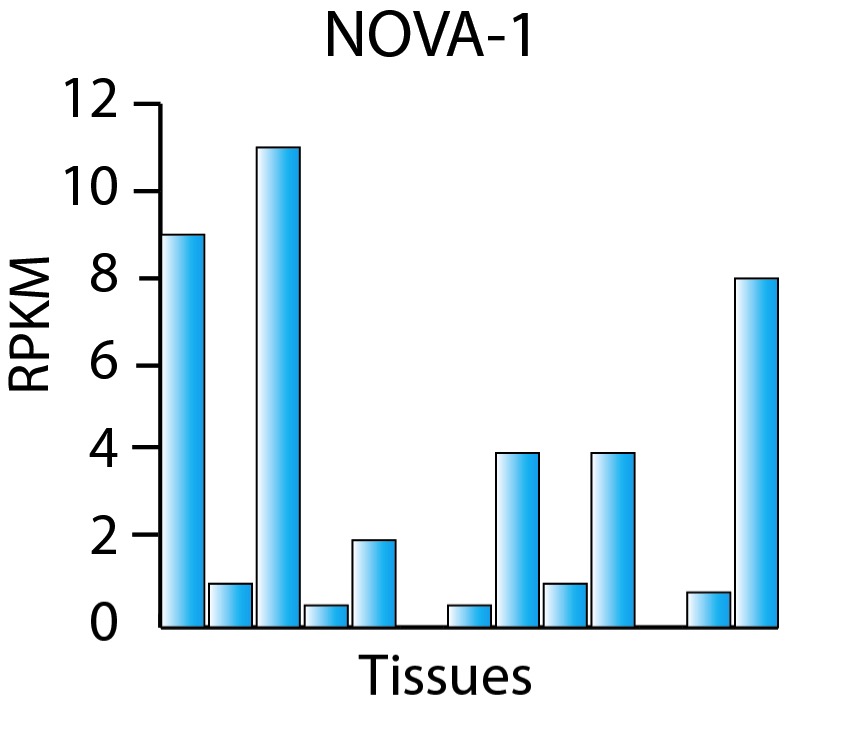
**

***Figure S4:*** *RNA expression profile for splice factor NOVA-1 in RNA mediated gene fusion transcript ENST00000446072, across a panel of human tissues on the x-axis (right to left): Adipose tissue; Adrenal gland; Brain; Heart; Kidney; Liver; Lung; Ovary; Pancreas; Sigmoid colon; Small intestine; Spleen, and Testis. Expression data is given in RPKMs. Expression data was obtained from the expression atlas ENCODE dataset (*Kapushesky et al, 2010)*.*

**Figure S5:** **Splice Factor Binding site profiles for fusion trancsript ENST00000567078 and it’s parent genes**

***Figure S6: A)*** *RNA-Mediated Gene Fusion transcript ENST00000567078 is displayed along with it’s parent genes RPS15A and ARL6IP1. Splice Fator binding sites for splice factor “SF2ASF” are represented by pink squares and for splice factor “NOVA-1” as blue squares.* ***B)*** *Expression level of Splice factor binding sites for splice factors SF2ASF and NOVA-1 for RNA-Mediated Gene Fusion transcript ENST00000567078 across a panel of human tissues on the x-axis (right to left): Adipose tissue; Adrenal gland; Brain; Heart; Kidney; Liver; Lung; Ovary; Pancreas; Sigmoid colon; Small intestine; Spleen, and Testis. Expression data is given in RPKMs. Expression data was obtained from the expression atlas ENCODE dataset (*Kapushesky et al, 2010)*.* ***C)*** *Expression profile of Splice factor binding sites of each of the parent genes ARL6IP1 (grey bars) and RPS15A (black bars). Tissue panel on the x-axis (left to right): Adipose tissue; Adrenal gland; Brain; Heart; Kidney; Liver; Lung; Ovary; Pancreas; Sigmoid colon; Small intestine; Spleen, and Testis. Expression data is given in RPKMs. Expression data was obtained from the expression atlas ENCODE dataset (*Kapushesky et al, 2010)*.*
